# Cytomegalovirus may influence vascular endothelial health in Indonesian HIV-infected patients after 5 years on ART

**DOI:** 10.1186/s12981-021-00410-7

**Published:** 2021-11-11

**Authors:** Ika Prasetya Wijaya, Birry Karim, Mohamad Syahrir Azizi, Ibnu Ariyanto, Arif Mansjoer, Evy Yunihastuti, Kuntjoro Harimurti, Idrus Alwi, Silvia Lee, Patricia Price

**Affiliations:** 1 Faculty of Medicine, Universitas Indonesia, Jakarta, Indonesia; 2 Cipto Mangunkusumo Hospital, Jakarta, Indonesia; 3 Virology and Cancer Pathobiology Research Centre, Faculty of Medicine, Universitas Indonesia, Jakarta, Indonesia; 4 School of Medicine, Curtin University, Bentley, 6102 Australia

**Keywords:** CMV, Endothelial function, HIV, Inflammatory biomarkers

## Abstract

**Objectives:**

Accelerated atherosclerosis in older HIV-infected patients has been attributed to persistent immune activation and high burden cytomegalovirus (CMV), as demonstrated in transplant recipients and the general population. Here we assess CMV and inflammatory markers linked with vascular health in young adult patients treated in Indonesia.

**Study design:**

HIV-infected adults (n = 32) were examined when they began antiretroviral therapy (ART) with < 200 CD4 T-cells/µl (V0) and after 60 months (V60). Age-matched healthy controls (HC, n = 32) were assessed once.

**Methods:**

Flow Mediated Dilatation (FMD) was assessed by ultrasound on brachial arteries at V60 and in HC. Plasma markers of immune activation and endothelial activation, and CMV antibodies (lysate, gB, IE-1) were assessed in all samples. Results were assessed using bivariate (non-parametric) and multivariable analyses.

**Results:**

Levels of inflammatory biomarkers and CMV antibodies declined on ART, but the antibodies remained higher than in HC. FMD values were similar in patients and HC at V60. In HIV patients, levels of CMV lysate antibody correlated inversely (r = − 0.37) with FMD. The optimal model predicting lower FMD values (adjusted R^2^ = 0.214, p = 0.012) included CMV lysate antibodies and chondroitin sulphate. In HC, levels of sTNFR correlated inversely with FMD (r = − 0.41) and remained as a risk factor in the optimal multivariable model, with CMV glycoprotein-B (gB) antibody predicting a healthier FMD (adjusted R^2^ = 0.248, p = 0.013).

**Conclusions:**

Higher levels CMV antibodies optimally predict vascular health measured by FMD in HIV patients. However in healthy controls, sTNFR marks risk and CMV gB antibody may be protective.

## Introduction

The wide availability of antiretroviral therapy (ART) has changed the profile of HIV disease from a life-threatening illness to one characterised by accelerated age-associated comorbidities, such as cardiovascular disease (CVD) [1]. However the relative contributions of traditional risk factors and HIV disease to vascular pathologies remains unclear, and there have been few studies in resource constrained or Asian populations. Without overt coronary heart disease, one can assess plaque and endothelial dysfunction through plasma biomarkers and non-invasive examinations. Meta-analyses establish increased risk of CVD in HIV-infected individuals based on carotid intima-media thickness (cIMT) and flow-mediated dilatation (FMD) [2], but the authors noted that many co-factors remain to be investigated. FMD assesses earlier stages of endothelium-dependent vasodilator function and has been validated as a surrogate of endothelial function of the coronary circulation, as it associates with prevalent and incident cardiovascular diseases [3]. FMD was independently predicted by high levels of antibodies reactive with cytomegalovirus (CMV) in renal transplant recipients [4]. CMV is addressed here in Indonesian HIV patients (the JakCCANDO study) with a very high CMV burden [5, 6]. In this population, changes to cIMT and cardiac parameters were small in the first year on ART [5].

CMV is a β-herpesvirus and replicates in endothelial cells, fibroblast and monocytes. Chronic infections can up-regulate leucocyte adhesion molecules (e.g. VCAM-1 and ICAM-1) and pro-inflammatory cytokines [7]. Recent meta-analyses establish links between CMV burden (assessed by seropositivity or levels of CMV-reactive antibodies) and CVD in the general population [8]. Hsue et al. [9] reported correlations between T-cell responses to CMV and increased cIMT in HIV patients in the US, and Knudsen et al. [10] described a direct association between levels of CMV-reactive antibodies and cIMT in Danish patients stable on ART. In our cohort, periodontitis and levels of CMV antibodies were independent predictors of raised cIMT after 5 years on ART [11].

Fewer studies have used FMD to assess HIV patients, although Parrinello et al. [12] reported an association between CMV antibodies and arterial stiffness in HIV-infected women. Andrade et al. [13] showed that FMD was significantly lower in Brazilian HIV patients, with a reduction on ART to values below healthy controls. CMV was not considered, and some groups have found no effect of HIV disease on FMD [14]. Here measures of the burden of CMV were evaluated alongside with markers of traditional markers of cardiovascular risk as determinants of FMD after 5 years on ART. We included Body Mass Index (BMI), but note that HIV patients with > 200 CD4 T-cells/µl are often overweight, while many patients with < 200 CD4 T-cells/µl are underweight [15, 16]. Levels of markers of inflammation (sTNFR-2, IL-6), endothelial activation (ICAM-1, VCAM-1) and coagulation (D-dimers) are higher in ART-naive HIV patients than in healthy controls, and may associate with accelerated atherosclerosis [17]. Chondroitin sulphate also plays a role in the modification and accumulation of lipids, especially low-density lipoprotein, and so may be pro-atherogenic in the general population with a role in early atherosclerotic lesions [18]. Patients undergoing endarterectomy to treat atherosclerotic plaque had higher plasma chondroitin sulphate levels than healthy controls [19]. We assess demographic parameters (age, gender, BMI), CD4 T-cell counts, biomarkers of inflammation and endothelial dysfunction (CRP, ICAM-1, sTNFR-1), chondroitin sulphate and CMV antibodies recognising Immediate Early (IE-1), lysate and glycoprotein B (gB) antigens.

## Materials and methods

### Subjects

JakCCANDO was a comprehensive survey of clinical and immunological responses in 75 HIV-infected patients who began ART with < 200 CD4 T-cells/µl in the HIV outpatient clinic of a tertiary hospital (Cipto Mangunkusumo Hospital, Jakarta, Indonesia) [5, 6, 11]. Here we describe a subset of 32 patients who attended a 5-year follow up (Visit60). Losses to follow up were attributed to increased access to clinics nearer their homes, changed phone numbers or home addresses, refusal to participate further and HIV-related death. Patients who returned and the 43 lost from the cohort were similar in all standard demographic parameters including age (32 ± 7.1 vs 32 ± 6.2 years, p = 0.63) and CD4 T-cell counts at V12 (304 ± 157 vs 332 ± 183 cells/µl, p = 0.26). Thirty-two healthy controls (HC) were assessed around the V60 timepoint, and matched the patient group by age and gender (see Table 1). The Health Research Ethics Committee Faculty of Medicine Universitas Indonesia & Cipto Mangunkusumo Hospital approved the study (no:133/UN2.FI/ETIK/2018) [5, 6].

**Table 1:** HIV patients maintained on ART for 5 years retain elevated responses to CMV, but levels of inflammatory biomarkers decline

	V0	V12	V60	Healthy controls
N	32	32	32	32
Age (years)	31 (27–36)	32 (28–37)	36 (32–41)	36 (32–43)
BMI (kg/m^2^)	19.7 (18.2–22.4)^b^	21.8 (19.9–24.4)^a,b^	22.9 (20.9–24.8) ^a,b^	25.3 (23.3–27.9)
Smoking [N (%)]				
Yes	10 (31)	–	13 (41)	8 (25)
No	12 (38)	–	16 (50)	18 (56)
Quit	10 (31)	–	3 (9)	6 (19)
CD4 T-cells/μL	61 (23–105)^b^	293 (171–430)^a,b^	459 (309–684)^a,^^b^	769 (594–1011)
Cardiovascular measures				
cIMT (mm)	0.58 (0.51– 0.64)^b^	0.58 (0.51– 0.70)^b^	0.56 (0.44– 0.70)	0.48 (0.45–0.59)
FMD (%)	–	–	7.42 (4.35–10.89)	8.33 (4.41–14.02)
Plasma biomarkers				
CRP (μg/mL)	1.01 (0.28– 4.24)^b^	0.79 (0.40–1.73)^b^	0.71 (0.22–1.62)	0.50 (0.13–0.77)
ICAM-1 (μg/mL)	0.41 (0.30–0.56)^b^	0.27 (0.21–0.37)^a,b^	0.24 (0.19–0.33)^a^	0.20 (0.17–0.25)
sTNFR (μg/mL)	0.42 (0.33–0.54)^b^	0.38 (0.31–0.43)^a^	0.35 (0.27–0.46)	0.32 (0.26–0.37)
Chondroitin sulphate (ng/mL)	69.5 (61–78)^b^	63 (58–69.5)^a,b^	54.5 (51.5–62)^a^	51.5 (48.5–59)
CMV-reactive antibodies				
CMV gB (AU × 10^–3^)	11.23 (3.99–29.87)^b^	15.42 (8.97–41.08)^a,b^	12.64 (8.21–2.15)^b^	8.19 (4.21–11.38)
CMV lysate (AU × 10^–3^)	20.77 (7.41–59.65)^b^	19.46 (6.58–50.05)^b^	18.54 (7.64–44.25)^b^	3.09 (1.99–5.80)
CMV IE-1 (AU × 10^–3^)	21.52 (10.63–49.15)	22.22 (11.02–57.29)	14.78 (9.43–33.24)	17.51 (13.16–24.61)

Visit (V) 0 represents the start of ART, V12 and V60 represent data collected after 12 and 60 months on ART. Continuous data are presented as median (interquartile range)

^a^Significantly different from V0, Wilcoxon test, *p* < 0.05

^b^Significantly different from healthy controls, Mann Whitney test, *p* < 0.05

### Carotid intima-media thickness (cIMT)

Carotid Doppler ultrasonography was assessed using an Esaote echocardiography machine and a LA522E probe (Genova, Italy) [4]. The cIMT measurements were performed by a single operator (BK) on greyscale 2D pictures, at sites with no focal lesions.

### Flow Mediated Dilatation (FMD)

Flow-mediated vasodilation was assessed in the brachial artery by ultrasonography (Esaote, probe LA522E) at V60. Participants were asked to abstain from exercise, caffeine, smoking or smoke exposure (≥ 12 h), vitamin supplementation (> 3 days), non-steroidal anti-inflammatory agents (1 day) and aspirins (3 days) and food (12 h). The measurements were performed on both arms with the patient in a supine position after 10–20 min rest. The brachial artery was scanned longitudinally above the antecubital crease using linear multi-frequency 5–12 MHz transducer B-mode. The diameter of the artery was measured on the interface between the media and adventitia of the anterior and posterior wall as a baseline. Gain settings were optimised to identify the lumen and the vessel wall interfaces and held constant. Hyperemia was induced by inflation of a pneumatic cuff on the proximal forearm to 200–250 mm Hg for 5 min. Arterial diameters were measured by one investigator (MS) 30–60 s before and after deflation of the cuff. Flow-mediated vasodilation was defined as 100 × [(post-hyperemia diameter − basal diameter)/basal diameter]. FMD values recorded in the right and left arms were correlated (HIV^+^: r = 0.49, p = 0.006; HC: r = 0.34, p = 0.059).

### Soluble TNFR, CRP, ICAM-1 and chondroitin sulphate

Plasma samples were aliquoted and stored at -80C. Soluble (s)TNFR-1, CRP and ICAM-1 were measured by ELISA using commercial antibody pairs (R&D Systems, Minneapolis, MN). Samples were serially diluted from 1:3000 for CRP, 1:100 for ICAM-1 and 1:10 for sTNFR [6]. Chondroitin sulphate was measured using a commercial ELISA kit (MyBioSource, San Diego, CA) with samples diluted 1:10.

### CMV Immediate Early (IE)-1, lysate and glycoprotein B (gB) antibodies

CMV-reactive IgG was quantified by ELISA. Parallel plates were coated with a lysate of human fibroblasts infected with CMV AD169, recombinant IE-1 (produced in *Escherichia coli,* Miltenyi Biotech; Cologne, Germany) and recombinant gB (kindly provided by Sanofi Pasteur; Lyon, France). Plasma samples were serially diluted from 1:10,000 (CMV lysate and gB) or 1:500 (IE-1). The binding was detected using goat anti-human IgG-horseradish peroxidase followed by tetramethylbenzidine substrate (Sigma Aldrich; Castle Hill, Australia). Antibodies were assessed relative to standard plasma pool assigned a value of 1000 arbitrary units (AU) [5, 6]. The method provides accurate quantitation in the high range.

### Statistical analyses

HC and HIV-infected patients were compared using non-parametric Mann–Whitney tests, whilst paired Wilcoxon tests were used to assess changes over time in patients. Natural log transformations (Ln) were used to approximate normal distributions where required and Pearson’s texts were used to identify bivariate associations between FMD (left arm) and patient characteristics, biomarkers and CMV antibodies. Multiple linear regression models were then initiated using all factors that achieved p ≤ 0.25 in the Pearson’s tests. Models were optimised by sequential elimination to identify factors that independently predict FMD. Regression analyses were performed in Stata SE 15.1 (StataCorp LP; College Station, TX). P-values < 0.05 are reported as significantly different.

## Results

### HIV patients maintained on ART for 5 years retain elevated responses to CMV

Smoking histories and age were similar in patients and HC. Patients’ CD4 T-cell counts and BMI increased on ART. Plasma biomarkers parameters generally decreased from V0 to V12 and V60, but were similar to HC at V60. Levels of antibodies reactive with CMV lysate and CMV gB remained elevated relative to HC (Table 1). Patients began ART with slightly higher cIMT values than HC, but FMD values were not significantly different from HC at V60.

### CMV lysate antibodies optimally predict FMD in HIV patients, whereas sTNFR predicts FMD in HC

**Fig. 1:**
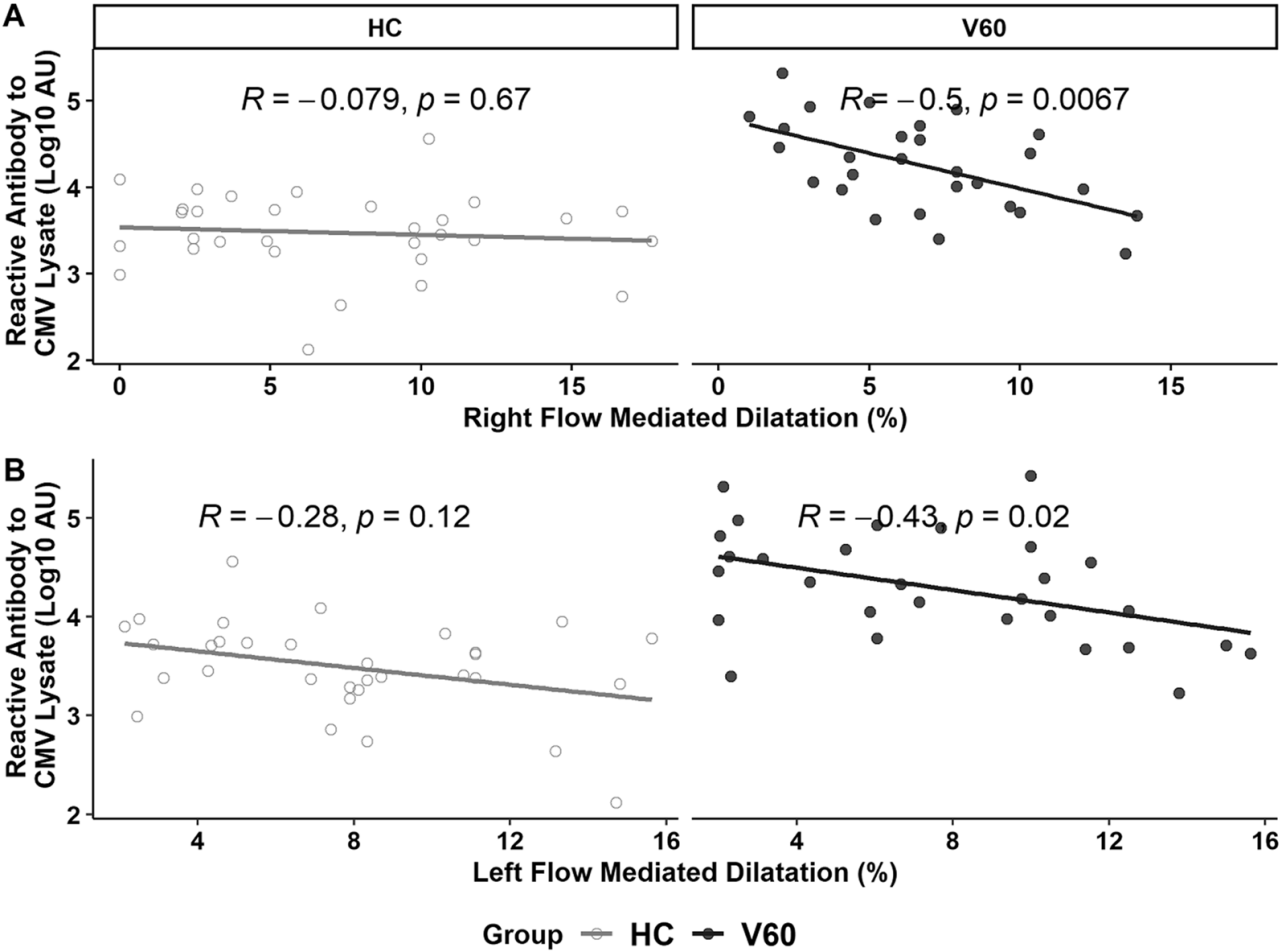
Levels of antibody reactive with CMV lysate were inversely related to FMD readings recorded in the right and left arms of HIV patients tested after 60 months on ART (V60) and not in healthy controls (HC). Spearman’s correlations are shown

Levels of CMV lysate antibody recorded at V60 showed a moderate inverse correlation with FMD at V60 in HIV-infected patients (p = 0.035, Table 2A, Fig. 1), linking robust response to CMV with poor vascular health. We found no other significant correlations between FMD and levels of any biomarkers assessed at V0 or V60, but a weak correlation was noted with levels of chondroitin sulphate at V60 (p = 0.110) so this was carried forward into multivariable analysis predicting FMD. Both remained significant in the optimal model (adjusted R^2^ = 0.214, p = 0.012). Data recorded at V0 did not provide a significant multivariable model predicting FMD at V60 (data not shown).

**Table 2:** Bivariate and mutivariable analyses link FMD with CMV antibodies at V60 in HIV patients, and with sTNFR in healthy controls

*Panel A*	HIV (V0)	HIV (V60)	HC
Age		− 0.18	− 0.06
BMI		− 0.005	− 0.32^b^
CD4 T-cells	− 0.09	0.010	0.09
Ln sTNFR	0.05	0.13	− 0.41^a^
Ln CRP	− 0.07	− 0.08	− 0.22^b^
Ln ICAM-1	− 0.003	0.07	− 0.26^b^
Ln CMV IE-1 antibody	− 0.19	0.17	0.06
Ln CMV lysate antibody	− 0.007	− 0.37^a^	− 0.14
Ln CMV gB antibody	− 0.17	0.02	0.21^b^
Chondroitin sulphate	− 0.06	− 0.29^b^	− 0.30^b^

Panel B	β coefficient	95% CI	P
Prediction of FMD in HIV patients at V60; Adjusted R^2^ = 0.214, p = 0.012			
Ln CMV lysate antibody	− 1.43	− 2.52 to − 0.34	0.012
Chondroitin sulphate	− 0.15	− 0.29 to − 0.01	0.035
Prediction of FMD in healthy controls; Adjusted R^2^ = 0.248, p = 0.013			
Ln sTNFR	− 7.31	− 13.04 to − 1.58	0.014
Ln CMV gB antibody	1.53	− 0.05 to 3.10	0.056
Chondroitin sulphate	− 0.16	− 0.37 to 0.05	0.136

Panel A presents Pearson’s r values assessing correlations with FMD

^a^ p value < 0.05

^b^p values of 0.05 to 0.25 were used to select parameters for the multivariable analyses

Panel B summarises the optimal multivariable models predicting FMD

For HC, plasma levels of sTNFR correlated inversely with FMD (p = 0.021; Table 2A) with weak inverse correlations with BMI (p = 0.074), chondroitin sulphate (p = 0.101), CRP (p = 0.247) and ICAM-1(p = 0.146) and not CMV lysate antibody (Fig. 1). We noted a weak direct association with CMV gB antibody (p = 0.247). These factors were included in our initial models. In the optimal model (Table 2B, adjusted R^2^ = 0.248, p = 0.013), sTNFR was most clearly associated with risk, but inclusion of chondroitin sulphate improved the model. In contrast, gB antibody appeared weakly protective.

## Discussion

Endothelial dysfunction is an early event in the development of atherosclerotic lesions and was assessed here after 5 years on ART using FMD of the brachial artery. FMD did not reveal endothelial damage attributable to HIV disease in our cohort. Previous studies have reported low or normal FMD values in patients on ART [2, 13, 14]. Most studies did not assess CMV but an association between FMD and CMV reactive antibodies was described in a largely middle aged male cohort based in California and stable on ART [20].

Here levels of CMV-reactive antibodies were extremely high in all patients ever tested in our clinic, being assayed from a starting dilution of 1:10,000. Levels decreased over 5 years of ART, but remained higher than in HC. Accordingly, in Australian patients commencing ART with < 210 CD4 T-cells/µL, levels of CMV antibody increased during the first year and remain elevated after 10 years [21]. The rise on ART suggests that levels of CMV-reactive antibody recorded before ART underestimate the high burden of CMV present at that time. The high CMV burden in JakCCANDO patients is evident from our finding that 50% of the cohort began ART with CMV DNA detectable with a simple in-house RT-PCR assay. This associated with low CD4/CD8 ratios and a depletion of naive T-cells and accumulation of memory T-cells, which may be a more stable metrics of the burden of CMV ^7^. Two patients retained CMV DNA detectable with this assay at V60 (in preparation).

Our key finding is that levels of CMV reactive antibodies on ART correlated inversely with FMD, as they do in renal transplant recipients [4]. The absence of associations between FMD and inflammatory biomarkers fits the observed decrease in these markers on ART to levels similar to healthy controls. The data suggest that CMV has a specific role in the pathogenesis of endothelial dysfunction that is not attributable to enhanced immune activation. It is probably relevant that levels of CMV reactive antibodies remain higher in patients than controls at V60, implying a higher viral burden.

FMD associated inversely with levels of sTNFR in healthy controls, with a weak protective effect of gB antibodies. gB also marked protection in our search for biomarkers that predict FMD measured in Australian renal transplant recipients after an interval of three years [22]. The association is plausible because CMV gB is considered a protective antigen based on its critical role in mediating viral-host cell fusion and thus viral entry. Phase II clinical trials of a monomeric recombinant trimeric CMV gB protein demonstrated efficacy in reducing viremia in solid organ transplantation recipients [23]. A corollary of our finding is that CMV itself is a negative influence in the HC, but is kept in check by a robust immune response marked by gB antibodies.

Chondroitin sulphate was included as a wild-card but was retained in the optimal multivariable models as it associated weakly with unhealthy FMD values in both patients and controls. This is accordance with the literature based on the general population [18, 19]. One can postulate a role in arterial stiffness but further studies should include assessments of other anionic polysaccharides. For example heparan sulfate interacts with HIV at a step prior to CD4 recognition, increasing infectivity by pre-concentrating the virion particles at the cell surface [24]. We have also demonstrated a role for heparan sulphate in murine CMV infection using cells pre-treated with heparinase [25]. However the present dataset shows no correlations between levels of chondroitin sulphate and CD4 T-cell counts (V0; r = -0.05: V60: r = 0.10) or levels of CMV lysate antibody (V0; r = 0.23: V60: r = − 0.05) and levels of chondroitin sulphate are similar in CMV DNA positive and negative patients when assessed at V0, V12 or V60 (p > 0.10). Further studies are needed, but this provides no evidence to link chondroitin sulphate with the burden of HIV or CMV.

We recognise the limitations of our study. The cohort is small and we did not assess plasma lipids or host genotypes. Moreover we did not assess FMD before ART, or assess CMV DNA at regular intervals to detect bursts of CMV replication. Nonetheless we show for the first time that higher levels CMV antibodies optimally predict vascular health measured by FMD in Indonesian HIV patients after 5 years on ART—individuals with a very high burden of CMV. The effect of CMV on FMD in patients was distinct from healthy controls.

## Data Availability

Data and relevant materials are available on request.

## Abbreviations

BMI: Body mass index
cIMT: Carotid intima-media thickness
CMV: Cytomegalovirus
FMD: Flow Mediated Dilatation
CRP: C reactive protein
ICAM-1: Intercellular adhesion molecule-1
sTNFR: Soluble tumour necrosis factor receptor
gB: Glycoprotein B
IE-1: Immediate Early-1
